# Antiviral use among hepatitis B infected patients in a low resource setting in Africa: a case study of West Nile, Uganda

**DOI:** 10.4314/ahs.v23i2.18

**Published:** 2023-06

**Authors:** Emmanuel Seremba, Claude Wandera, Richard Ssekitoleko, Joan Nankya-Mutyoba, Filbert Nyeko, Jacinto Amandua, David Ejalu, William Omale, Ponsiano Ocama

**Affiliations:** 1 School of Medicine, Makerere University College of Health Sciences; 2 Infectious Disease Institute, Makerere University College of Health Sciences; 3 School of Public Health, Makerere University College of Health Sciences; 4 Arua Regional Referral Hospital; 5 Republic of Uganda Ministry of Health

**Keywords:** Hepatitis B Virus, treatment, Uganda

## Abstract

Failure to access antiviral medications is a leading cause of hepatitis B (HBV)-associated morbidity and mortality in sub-Saharan Africa (SSA). Despite guideline availability, SSA is not on course to meet its elimination targets. We characterized factors associated with antiviral medication use and challenges to offering chronic care in a large Ugandan institution. We abstracted HBV care data. 2,175/2,209 (98.5%) had HBV-infection. Most participants were men [1,197 (55%)]; median (IQR) age 27 years (19-35); 388/1689 (23.0%) had cirrhosis by sonography and 141/2175 (6.5%) by the aspartate aminotransferase to platelet ratio index (APRI) score ≥2. Of the eligible, 20/141 (14.2%) with APRI score ≥2 and 24/388 (6.2%) with sonographic evidence of liver cirrhosis were not on antiviral medications. Overall, 1,106 (51%) were on medications though 65.8% had not been fully investigated. In multivariate analysis, age ≥35 years [OR (95% CI) = 1.52 (1.01-2.28), p=0.043], APRI ≥2 [OR (95% CI) =1.79 (1.482.16), p<0.001], hepatitis B viral load >2,000IU/mL [OR (95% CI) = 6.22 (5.08-7.62), p<0.001] were associated with antiviral medications use. Over half of participants in care had not been fully evaluated although on treatment and many eligible patients did not access medications. There is need to bridge these gaps for SSA to realise its HBV elimination goals.

## Introduction

Recent data suggest that the annual mortality from viral hepatitis B and C exceeds that of Human Immuno-deficiency virus (HIV) and is comparable to that of tuberculosis^1^. In 2015, viral hepatitis B and C are believed to have caused 1.34 million deaths from complications of cirrhosis and hepatocellular carcinoma (HCC) 1. Despite the availability of effective vaccines and antiviral medications, 887 000 of these deaths were attributed to the hepatitis B virus (HBV) 2. The majority of these deaths occurred in sub-Saharan Africa (SSA) where 60 of the 257 million that are infected with HBV reside 1.

Uganda is moderately endemic for HBV, with a national prevalence of the hepatitis B surface antigen (HBsAg) positivity of about 4.1%. This prevalence however varies widely being highest in the Mid-North (4.6%) and lowest In the Southwest (0.8%) 3. In response to this threat, in 2002, the Uganda government through its childhood expanded programme on immunization introduced the HBV vaccine, which is administered free of charge, in form of a pentavalent vaccine at 6, 10 and 14 weeks.

Cognisant of the HBV-related morbidity and mortality toll, the World Health Organization (WHO) in its 63rd World Health Assembly spells out the seriousness of viral hepatitis and expresses concern on the lack of progress in the prevention and control of viral hepatitis in developing countries especially in the SSA region. In this resolution, the lack of access to affordable, appropriate treatment and care as well as an integrated approach to the prevention and control measures of the disease is stressed 4.

These are further emphasized in their next resolution on hepatitis (2014) which seeks to eliminate this disease as a public health threat by 2030 and the Global Health Sector Strategy on Viral Hepatitis 2016-2021 5,6.

In line with these resolutions, over the last five years, Uganda has been re-training non-specialist physicians (medical doctors) with a long-term goal of decentralizing and integrating hepatitis care in the existing service package. The campaign was planned to be performed in a phased manner, starting with the most highly burdened regions in the country, but to eventually cover the whole country. The West Nile region, with a prevalence rate of 3.1% was the first to host the campaign. Announcements were made through various media platforms inviting the population to go to designated health care centres in the region for testing using rapid strip assays. All those who were found infected were then referred to the regional referral hospital for further evaluation and care. To date close to 4 million adults in the country have so far been screened for the virus and over 250,000 found to be infected. Antiviral medications particularly tenofovir have also been availed to Regional referral hospitals for the treatment of eligible patients 7.

Uganda uses a National Guideline for Prevention, Testing, Care and Treatment of Hepatitis B and C Virus Infection 8 that was adapted from the 2015 WHO Guidelines for the prevention, care and treatment of persons with chronic hepatitis B infection 9. Briefly, this guideline considers: decompensated cirrhosis, HIV co-infection, aspartate aminotransferase to platelet ratio index (APRI) in assessing patients for eligibility to commence on anti-viral medications for hepatitis B infection.

To facilitate the identification of patients that meet the eligibility criteria for antiviral medications, the Uganda Government through its Central Public Health Laboratories has been offering free quantitative HBV viral load valuation, with each patient being eligible to at least one round of testing every year. It also provides tests for HIV, liver function, renal function as well as a complete blood count at health facilities (Referral and General hospitals) that are currently mandated to treat HBV infection.

Despite achieving the above strides in HBV prevention and care, Uganda still strives to link screened patients to chronic care and to offer antiviral medications to the eligible individuals in time. To highlight the treatment-related factors and challenges, we conducted a retrospective study at one of the national centres of excellence for HBV care at a rural Regional referral hospital in Uganda, where HBV-related diseases constitute a sizeable proportion of the disease burden- where on average 3.7% of the population has chronic HBV 3. The disease burden in the Northern region of which West Nile is part is however higher in some other subpopulations including persons living with HIV and the pregnant women where HBV is prevalent in over 11% of these individuals 10-12.

## Methods

This was a study conducted in Arua district, in the West Nile region of Uganda in which we obtained data from the Arua District Health Office as well as the Arua Regional Referral Hospital (ARRH). The District Health Office is mandated to coordinate health promotion, preventive and curative services between the central Government Ministry of Health and the health facilities within its area of responsibility. It thus houses a dedicated records centre where summarized data on utilization of the above services in the district can be accessed. From this office, we obtained aggregated data from a previously Government-spearheaded mass public hepatitis B screening exercise as well as information on the other health facilities (other than ARRH) that offer HBV- care services and the number of individuals that utilize this service.

The ARRH serves as the district hospital for Arua, as well as the regional referral health care facility for the West Nile region of Uganda. By virtue of its location, it also serves the neighbouring populations from South Sudan and the Democratic Republic of Congo. It offers specialist services including a dedicated hepatitis B clinic which has been in operation since 2014 under the headship of a specialist Physician. Prior to this (2010-2014), the hospital had been offering care to HBV-infected patients in a general medical clinic setting. This clinic runs once a week and attends to both children and adults with HBV mono infection. Those that are coinfected with HIV and HBV are treated in the HIV clinic.

In this retrospective study, medical records of patients attending the above clinic over a 5- year period (2014-2019) were retrieved and data relevant to HBV management were abstracted and entered in an excel spread sheet. The variables captured from these charts included; social demographic variables- age at registration in the clinic, sex; laboratory parameters- hepatitis B surface antigen test (HBsAg) result, the most recent Human Immunodeficiency Virus (HIV) status, liver enzyme (ALT, AST) levels, platelet count, hepatitis B e antigen (HBeAg), hepatitis B viral load levels; alpha feto protein (AFP) levels; abdominal ultrasound scan findings; evidence of decom-pensated liver disease (ascites, hepatic encephalopathy, upper gastrointestinal tract bleeding) and use of antiviral medications (tenofovir monotherapy or a combined pill of tenofovir and lamivudine). This combined pill is used when the hospital runs low on TDF and is also the recommended treatment approach (together with another anti-retroviral medication) for HIV/HBV co-infected patients. We excluded individuals whose HBsAg or hepatitis B viral load test results were missing and those that were coinfected with HIV, as their management is by policy conducted in the HIV clinics.

Using the above data (liver enzymes and platelet count), we assessed the eligibility of our study participants for antiviral medications by computing their APRI score using the formula APRI= (AST/ULN) x 100) / Platelet count (109/L). Basing on the WHO guideline, a pre-defined score of ≥2 was used as a surrogate for cirrhosis and thus eligibility for medications 9. Prior to inauguration of the national guideline, the majority of clinicians relied on the WHO and some on the European and American liver Associations guidelines which on top of the liver enzymes and HIV tests, also require liver biopsy/histology 13, 14 – which is not readily available in resource limited settings for patient management.

The outcome of this study was defined as use or non-use of antiviral medications for HBV.

### Statistical analysis

Data were exported from the excel sheet and analysed using STATA software package, version 14.0 (College Station, Texas, USA). Descriptive characteristics of the participants were summarized. Categorical variables were evaluated by means of proportions, while medians (inter-quartile range) or means (standard deviation) were obtained for the continuous variables.

Patients on antiviral medications were expressed as a percentage of those that were eligible for treatment based on the APRI score criterion and on liver sonography. The association of the different variables to the use of antiviral medication was assessed. Chi-square tests were used to assess the association of categorical variables to the outcome while logistic regression was used to assess the association of the continuous variables to the outcome. Variables with a p-value <0.2 were included in multivariable logistic regression. Results of the association of the variables to the outcome were summarized as odds ratios, with 95% confidence intervals and p values. Statistical significance was considered at a 2-sided p-value of ≤0.05. This study was approved by the School of Medicine Makerere University College of Health Sciences Research and Ethics Committee (SOMREC) (approval REC Ref 2018-185) and the Uganda National Council of Science and Technology (UNCST) (approval Ref SS 4986). Administrative clearance was granted by both the district health office and the hospital administration.

## Results

### HBV disease burden and chronic care in the district

Records from the district health office revealed that in the mass HBV screening exercise, 218,408 adults were tested and 14,176 (6%) found to be infected with this disease. Besides ARRH, only one other health facility, a private not for profit hospital, was involved in HBV care. A total of 150 HBV-infected patients were found to be accessing chronic care at this facility.

### Chart review at HBV Clinic, ARRH

**Figure 1:**
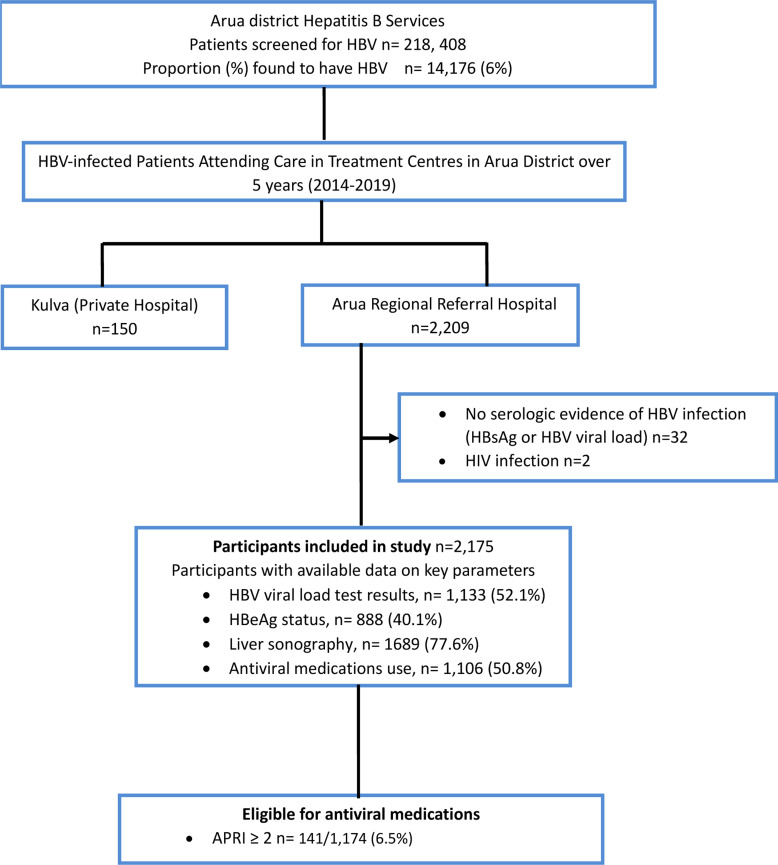
Flowchart of selected participants HBeAg = hepatitis B e antigen, APRI= aspartate aminotransferase to platelet ratio index, Antiviral medications= tenofovir monotherapy or tenofovir in combination with lamivudine. Of the 1689 that had abdominal ultrasounds scans, 23.0% (388/1689) features of cirrhosis

Of the 2,209 individuals whose charts were evaluated, 32 were excluded either because their HBsAg or viral load tests were missing or were coinfected with HIV (n=2). 2,175 were therefore eligible for the study and were included in the study. They were predominately male 1,197 (55%), and young, with a mean (SD) age of 28.6 (12) years (table 1). Approximately a half (1,133, 52%) of the participants had HBV viral load test results, geometric mean (95% CI) =110,340.30 (80,402.22-151,425.80); 388 (17.8%) of the participants had sonographic evidence of liver cirrhosis and 141 (6.5%) had evidence of liver fibrosis (APRI score >2). About a half (1,106, 51%) of the participants had received antiviral medications (Figure 1), 65.8% of whom had not been fully investigated. Antiviral medication data were not available for 11 (0.5%) participants. These were excluded from the analysis of the study outcome (antiviral medication use).

**Table 1:** Descriptive characteristics of the study population

Characteristic	N (%)
Sex ^a^	
Females	900 (41.40)
Males	1,197 (55.06)
	
Age years (mean (SD)	28.6 (12)
	
HBeAg ^b^	
Negative	593 (27.31)
Positive	295 (13.59)
	
Ultrasound Scan ^c^	
Normal	1,301 (59.84)
Evidence of cirrhosis	388 (17.85)
	
Antiviral therapy (tenofovir) use ^d^	
Yes	1,106 (50.87)
No	1,057 (48.62)
	
ALT (IU/Ml) ^e^	
≥2XULN	219 (10.07)
<2XULN	1,589 (73.09)
	
AST (IU/Ml) ^f^	
≥2XULN	310 (14.26)
<2XULN	1,487 (68.40)
	
APRI score ^g^	
<2	906 (41.69)
≥2	141 (6.49)
	
Hepatitis B viral load	
Detectable	1,133 (52.12)
Missing	1,041 (47.88)
Below detection limit	137 (6.30)
HBV Viral load (IU/Ml) ^h^	110,340.30 CI (80,402.22- 151,425.80)

**Missing data:** a Gender 77 (3.54%), b HBeAg 1,283 (59.10%), c abdominal ultrasound scan 485 (22.31%), d Anti-viral therapy use 11 (0.51%), e ALT 366 (16.84%), f AST 377 (17.34%), g APRI score 1,126 (51.82%). h Geometric mean and 95% confidence intervals of 996 hepatitis B-infected individuals whose viral load was above the lower limit of detection of the assay.

### Eligibility for antiviral medications

Altogether, 6.5% (141/2175) participants were eligible for antiviral medications basing on the APRI score ≥2 and of these 121/141(85.8%) had been initiated on them. In this study sample, 77.6% (1689/2,175) had an abdominal ultrasound scan performed reporting on the liver; of these, 22.3% (388/1689) showed features suggestive of cirrhosis and 93.8% (364/388) were on antiviral medications. Of note, 257/1106 (23.2%) of the participants with APRI score <2 was on antiviral medications.

Only 11/2175 (0.5%) participants had ever been screened for HCC using the AFP biomarker. Of those that had this biomarker and additional evaluation, 3/7 that had liver sonography had evidence of cirrhosis, 5/6 in whom HBV viral load results were available had high HBV viral load levels ranging from 220,000-150,000,000 IU/mL, 4/5 in whom HBeAg were recorded had HBeAg positive disease and a total of 9/11 were on antiviral medications. In univariate analysis, male gender [OR (95% CI) = 1.58, (1.33-1.88), p <0.001], age ≥35 years [OR (95% CI) = 1.28 (1.04-1.56), p=0.017], positive HBeAg chronic HBV [OR (95% CI)=0.88 (0.80-0.97), p=0.013] cirrhosis (by liver sonography) [OR (95% CI)=23.22 (15.14-35.62), p <0.001], APRI score ≥2 [OR (95% CI) = 15.28 (9.3225.05), p <0.001] were found to be associated with use of antiviral medications. Similar to increasing age, a higher HBV viral load was also associated with use of antiviral medications. Among individuals with HBV DNA levels of 2,000-19,999IU/mL, the odds of using antiviral medications were 18.04 times the odds for those with HBV DNA levels <2,000IU/mL CI (11.37-28.63) and much higher, 36.30 times CI (25.32-52.03), P<0.001] in those with ≥ 20,000IUmL as compared to those with a viral load <2,000IU/mL (table 2).

**Table 2:** Univariate analysis of factors associated with use of antiviral medications

	Odds Ratio (95% CI)	P-value
Sex		
Women	Ref	**<0.001**
Men	1.58 (1.33-1.88)	
		
Age		
<35	Ref	**0.017**
≥35	1.28 (1.04-1.56)	
		
HBeAg		
Negative	Ref	**0.013**
positive	0.88 (0.80-0.97)	
		
Ultrasound scan findings		
Normal	Ref	
Cirrhosis	23.22 (15.14-35.62)	**<0.001**
		
APRI score		
< 2	Ref	**<0.001**
≥2	15.28 (9.32-25.05)	
		
Hepatitis B viral load (IU/mL)		
Undetectable -1,999	Ref	**<0.001**
2,000-19,999	18.04 (11.37-28.63)	
≥20,000	36.30 (25.32-52.03)	

In multivariable analysis, only age ≥35 years [OR (95% viral load levels >2,000IU/mL [OR (95% CI) = 6.22 CI) =1.52 (1.01-2.28), p=0.043], APRI score ≥2 [OR (5.08-7.62), P<0.001] were associated with use of antivi-(95% CI) = 1.79 (1.48-2.16), p<0.001], and hepatitis B ral medications (table 3).

**Table 3:** Multivariable analysis of factors associated with antiviral medication use

	Odds Ratio (95% CI)	P=value
Sex	1.05 (0.75-1.47)	0.766
		
Age>35 years	1.52 (1.01-2.28)	**0.043**
		
Hepatitis B e Ag	0.93 (0.77-1.13)	0.479
		
Cirrhosis (by liver sonography)	1.20 (0.97-1.48)	0.096
		
APRI score ≥2	1.79 (1.48-2.16)	**<0.001**
		
HBV viral load >2000IU/mL	6.22 (5.08-7.62)	**<0.001**

## Discussion

According to the Center for Disease Analysis Foundation (CDAF), a life is lost due to hepatitis B-related complications every 2.5 minutes in SSA 15. This high mortality is undoubtedly a consequence of poor access to vaccination and anti-viral medications in this region.

We found that less than 20% of the 14,176 persons that were diagnosed with HBV in a mass screening campaign in Arua district 7 accessed chronic care, with the majority 2,209 (15.6%) attending at ARRH, the largest and best equipped health facility in the district that takes care of HBV-infected persons, and only a smaller proportion 150 (1.06%) sought care at the alternative service provider in the district. These being the facilities that take care of almost all the HBV patients in chronic care in the district implies that over 80% of those diagnosed with this disease on screening are lost to follow up, most probably due to gaps in linkage to care. Given that the hospital had been providing HBV care prior to the mass screening, a considerable number of patients attending the ARRH HBV clinic may not necessarily be clients from the mass screening exercise which took place much later (20152016) in the district. This gap in linkage to care is in agreement with an earlier study in Burkina Faso where 75% of HBV-infected patients never made it into care, citing the lack of resources, infrastructure and trained health care providers, preference for traditional healers among the barriers to linkage 16. This underscores the need to scale up efforts to overcome these and other barriers as linkage to care is pivotal to a successful hepatitis care sub-programme in SSA 17.

The majority 121/141 (85.8%) of our study participants that were eligible for antiviral medications based on the APRI score> 2 were on treatment. Of concern 14.1% (20/141) who were eligible by the same criteria and 24/388 (6.2%) with sonographic evidence of liver cirrhosis- a key driver of liver-related morbidity and mortality had not been initiated on antiviral medications. This threatens the survival of these patients as they are in immediate need of antiviral therapy. With emerging data showing that at the traditional APRI score cut-off of ≥2 a significant number of patients with cirrhosis/ significant liver fibrosis may be missed 18, 19. Thus, the proportion of our study population that is in urgent need of these medications could be much higher. This may explain why some studies opted to use a lower APRI score cut-off 20. This diagnostic challenge is coupled with lack of funding for HBeAg and liver histology. It highlights a need for further studies to refine the APRI score criterion and to identify other additional or stand-alone less-costly approaches to detecting liver fibrosis in low resource settings of SSA at an early stage. Investing in liver elastography that could facilitate use of the rather more accurate modified Western criteria 21-23 could also be a worthwhile option in countries like Uganda where government offers free HBV load testing to patients with chronic disease. In the meantime, training more health care providers and decentralizing disease management to lower health facilities would relieve the huge work load on the existing low staffing levels. In the interim, a more workable solution to minimize missed opportunities for initiating medications to eligible persons would perhaps be achieved by integrating the HBV with the HIV/AIDS or other existing disease care services.

Over a half of our participants (51%) were on antiviral medications. This is exceedingly higher than the estimated 12-25% that should be eligible for these medications in cohorts of patients with chronic HBV infection^24^. Of interest, 23.2% of individuals on antiviral medications had an APRI score <2 and would not be eligible for treatment by the WHO/local guideline. These are likely to have been initiated on treatment when neither of these treatment guidelines was in place and health care providers had not been trained on disease management. It would therefore be understandable that knowledge gaps among care providers on various aspects of disease management including the eligibility criteria for initiating antiviral medications were high.

The very high proportion on treatment could have been a consequence of late presentation with decompensated cirrhosis for care; however decompensated cirrhosis being a relatively rare presentation, and with the least expensive guide to determining eligibility for antiviral medications, the APRI score, missing in over 50% of study participants due to lack of either a complete blood count, liver function tests or both, the clinicians' ability to identify patients that were eligible for treatment as per the treatment guideline was undoubtedly impaired.. Indeed, 65.8% on antiviral medications had not been fully investigated. The provision of the above tests in public health facilities is dictated by the availability of funds. However, test results may still be unavailable especially in charts of those that source them from private facilities as they at times opt to take custody of their laboratory test reports. This suggests a need to improve documentation and records keeping.

Since HBV-related HCC may be minimised by timely initiation of antiviral medications, we opted to assess antiviral use at cut-off age of 35 years so as to get an insight of antiviral medication use with reference to the age when the HCC incidence in SSA becomes an apparently big concern 25. We found a significant increase in tenofovir use with increasing age, a promising trend in reducing HCC. Recent data from a Gambian longitudinal cohort of patients with chronic HBV showed that the age-specific incidence of HCC in male chronic HBV carriers increased with age, being 0.0, 304.2, 634.6, 563.3 and 1538.1 per 100,000 carrier-years for those 25-34, 35-44, 45-54, 55-64 and ≥65 years, respectively^25^. In addition, the significant trend of increasing odds of antiviral use with increasing HBV viral load that we observed at hepatitis B viral load of 2,000 and 20,000IU/mL is in agreement with clinical practice in the Western world where HBV care guidelines recommend initiation of antiviral medications to chronic HBV-infected individuals at these cut-off points^13^, ^14^.

Screening individuals with chronic HBV for HCC should be part of routine care. In developed countries, this is usually achieved by biannual ultrasound scans and at times in combination with measuring of the AFP. In our study, the use of serial ultrasound scans for this purpose was not evaluated. We however noted that only 0.5% of our participants had been screened for this tumour using AFP measurements. This is not surprising in Uganda and many other settings in SSA where there is scarcity of HCC treatment modalities that can potentially prolong life even when the tumour is diagnosed at an early stage when a cure can still be achieved.

Our study has some limitations. We used data from a single centre that was not originally designed to answer our research question; thus, we had missing and incomplete data on some study parameters such as decompensated liver disease and serial liver enzyme levels to ascertain persistently elevated ALT levels which in combination with HBV viral load levels of >20 000 IU/mL would constitute other criteria for antiviral medication use. Similarly, data on potential confounders particularly alcohol misuse and schistosomiasis were not captured. In addition, the selection of the charts was non-random and this could have led to selection bias. Also, the year of initiation of antiviral medications that would enable us to correct for changes over time was not captured. Further, the temporal association of the measured variables to the initiation of antiviral medications could not be ascertained. However, we did access a large volume of data from a centre of excellence for HBV management in Uganda, a WHO model country for HBV care in SSA. Our study findings therefore offer a good insight into factors associated with medication use and the challenges that health facilities that take care of HBV in resource poor settings of SSA are likely to face.

## Conclusion

A high proportion of patients that are screened for HBV-infection don't make it to health care facilities for chronic care. In addition, many patients in chronic care are not fully evaluated for eligibility for treatment. Even among those that are eligible for antiviral medications including those with cirrhosis, a sizeable proportion is not receiving them.

These findings demonstrate the need for public health education, infant vaccination, research into the barriers to linkage, strengthening the linkage to care, evaluation and treatment of HBV-infected patients in low resource settings if the WHO-driven goal to eliminate HBV as a public health threat by 2030 is to be realized. Integrating HBV and HIV care services could be a viable option.
